# The History and Faults of the Body Mass Index and Where to Look Next: A Literature Review

**DOI:** 10.7759/cureus.48230

**Published:** 2023-11-03

**Authors:** Rachel Pray, Suzanne Riskin

**Affiliations:** 1 Medicine, Nova Southeastern University Dr. Kiran C. Patel College of Osteopathic Medicine, Clearwater, USA; 2 Internal Medicine, Nova Southeastern University Dr. Kiran C. Patel College of Osteopathic Medicine, Clearwater, USA

**Keywords:** waist-to-height ratio, waist-to-hip ratio, abdominal adiposity, ancel keys, body mass index

## Abstract

Body mass index (BMI) is an anthropometric index that is commonly used in the medical setting and is a factor in assessing various disease risks but its origins are unknown by many. More importantly, BMI does not properly assess body fat percentage and muscle mass or distinguish abdominal fat from gluteofemoral fat, which is important to note because abdominal fat is associated with insulin resistance, metabolic disease, and cardiovascular complications. Using a less accurate index to assess the relationship between weight and disease risk is conceptually invalid because the use of BMI ultimately trickles into patient treatment, preventive medicine, and overall health outcomes. Several different anthropometric indices that more accurately assess abdominal adiposity through the incorporation of waist circumference exist and have been extensively studied, such as waist-to-hip ratio, waist-to-height ratio, and a body shape index. It is important that we consider replacing BMI’s usage in the healthcare setting with a different anthropometric index: one that considers height, sex, and race differences, accounts for abdominal adiposity, and more accurately predicts the relationship between obesity, mortality, and diseases such as cardiovascular disease, hypertension, insulin resistance, and diabetes.

## Introduction and background

The history of BMI dates back to 1832. Adolphe Quetelet (1796-1874), a Belgian statistician, mathematician, and astronomer, was inspired by his passion for statistical analysis and bell-shaped curves to establish quantifiable characteristics of the "normal man." Thus, the Quetelet Index was born: weight divided by height squared [1]. In the 1950s, Louis I. Dublin (1882-1969), statistician and vice president of Metropolitan Life Insurance Company, established tables of normal weights for clients, after the company noted that more and more claims were coming from their obese policyholders [1]. Dublin's tables did not specify age, though they did place clients into three categories based on their frame: “small,” “medium,” and “large” [1,2]. In 1972, physiologist Ancel Keys (1904-2004) built upon the work of Adolphe Quetelet’s Quetelet Index. He officially coined the modern term “body mass index” and provided evidence to support its current usage with his 1972 study [3], in which he analyzed 12 samples of 7,426 "healthy" men. Keys emphasized the convenience and ease of using BMI in population studies to analyze data [2]. In 1997, the World Health Organization consultation formally recognized obesity as a global epidemic [4]. BMI now exists on almost all electronic medical records (EMR), is used as a factor in assessing various disease risks, and is commonly known to most adults and children in the US [5].

Materials and methods

Several procedures were carried out to obtain a quality, comprehensive review of the literature on BMI. First, a literature search and screening were performed by searching through three databases: PubMed, Embase, and Access Medicine. The first search was performed on PubMed using the terms, "body mass index," "history of body mass index," and "cardiovascular disease," uncovering 7,616 results. The second search was performed using Embase, including the terms, "body mass index," "gender differences," "cardiovascular disease," and "waist to hip ratio," uncovering 96 results. A third search performed on Embase included the terms "body mass index," "racism," and "cardiovascular disease," uncovering 36 results. A fourth search on Access Medicine was performed using the search term "body mass index," uncovering 1,235 results. In total, the search process uncovered 8,983 peer-reviewed articles published from 2000 to 2021. Of the 8,983 articles uncovered, 44 articles were thoroughly screened. Inclusion criteria consisted of articles discussing the history of BMI; studies with the main objective of examining BMI in populations of various backgrounds and sexes; studies that examined the relationship between a combination of the following: BMI, cardiovascular disease, hypertension, and metabolic disease; and studies that analyzed the use of varying anthropometric indices in various populations. Exclusion criteria were based on minimal or no mention of BMI and studies that did not isolate the impacts of BMI adequately. The reference section for these articles was also searched to find additional articles, thus, some papers were found from end-of-publication citations. Data extraction and analysis were ultimately performed on 27 articles. These 27 articles contributed to this literature review and are cited in the references section.

## Review

Results

The Importance of Considering Abdominal Adiposity

Abdominal fat, also known as central obesity, accounts for fat around the waist and is associated with insulin resistance (IR), metabolic disease, and cardiovascular complications [6]. Gluteofemoral fat accounts for fat around the hips and is normally higher in women [7]. Due to the significant relationship between abdominal/overall obesity and coronary heart disease mortality risk/cardiometabolic risk, it is more clinically important to assess abdominal fat compared to gluteofemoral fat in most cases [8,9].

Larger sagittal abdominal diameter (SAD) was associated with a significantly increased risk of sudden death in asymptomatic French middle-aged men, independent of BMI and known cardiovascular disease (CVD) risk factors. The sagittal abdominal diameter measured below the xiphoid (SADx), not BMI, was associated with a significantly increased risk of fatal myocardial infarction and sudden death when the same study introduced SADx and BMI into the same multivariate regression Cox model [10]. Although it is still extremely widely used, BMI is unable to accurately predict abdominal obesity [8]. BMI also does not distinguish between muscle mass and adipose tissue [11]. In a prospective cohort study of 44,636 women in the Nurses’ Health Study, Zhang et al. concluded that independent of BMI, anthropometric measures of abdominal adiposity were significantly and positively associated with CVD and cancer mortality. Abdominal obesity was significantly associated with elevated CVD mortality in normal-weight women [9]. Of the various anthropometric indices used in the study - waist circumference (WC), waist-to-hip ratio (WHR), waist-to-height ratio (WHtR), hip circumference (HC), height, and BMI - both WC and WHR were strongly associated with mortality in women who were not overweight or obese [9].

The 1972 Ancel Keys' Study and Its Major Limitations

BMI was originally established in the 19th century by a statistician and rebranded in the 21st century by a physiologist for capitalist and research benefits [1,2]. Neither of these individuals were medical professionals.

Keys did not intend on implementing BMI for medical use; he was a physiologist intrigued by the human body and its statistical analysis. He described it as a simple, obtainable measurement that translated with ease to the research setting. In his study, he acknowledges the superiority of body density in assessing body fat mass but states that it is not feasible for routine or survey use due to its time-consuming nature. Keys states, “the best relative weight index is the one that shows the least correlation with body height and the highest correlation with independent measures of body fatness.” He admits that “still, if density is truly and closely (inversely) proportional to body fatness, not more than half of the total variance of body fatness is accounted for by the regression of fatness on the body mass index” [2]. We cannot continue to ignore the fact that the original study that sparked BMI’s popularity in everyday life - and ultimately into the medical setting - admitted that it is not the best indicator of body fat mass.

Along with BMI’s fundamental limitation - its inability to accurately measure body fatness - other limitations existed within Keys’ study. The ages of the cohorts of men were not uniform. The “Bantu” cohort consisted of men aged 31-60 years, the “University of Minnesota students” cohort consisted of men aged 18-24 years, the “Minnesota executives” cohort consisted of men aged 49-59 years, and all other cohorts consisted of men aged 40-59 years. No women were included in the studies and these studies were not properly representative of different ethnicities, races, and backgrounds. Of the 7,426 men included, just 1.56% of them were of South African descent, from the “Bantu” cohort; 13.9% were of Asian descent from the “Japanese farmers” and “Japanese fishermen” cohorts; 43.7% of men were of European descent from “East Finland,” “West Finland,” “Crevalcore, Italy,” and “Montegiorgio, Italy”; 40.78% of the participants were men from the United States from the “Rome Railroad,” “University of Minnesota students,” “Minnesota executives,” “U.S. Ry. sedentary,” and “U.S. Ry., switchmen” cohorts [2]. While there was some representation of different ethnic groups in the 1972 Keys' study, this representation was somewhat random and was greatly outweighed by that of Caucasian men of European descent. It would have been beneficial to use a greater number of men from Asian and African backgrounds, along with including Hispanic men, to provide more accurate data regarding the use of BMI in these populations and to be more representative of the population of the United States, and different parts of the world. Due to the smaller amount of subjects from groups that were not from the United States or Europe and no representation of women subjects in this study, BMI may not have been an accurate assessment of adiposity for individuals of various races, ethnicities, backgrounds, and sexes.

Discussion

Racial and Ethnic Considerations

It is time to introduce a new anthropometric index of weight inclusive to women and minorities that is a more accurate predictor of disease risk, has a stronger relationship between body fat percentage (BF%) and adiposity, and is a better representation of our patients’ overall health. Women and minorities were not included at all, or only comprised a small percentage of the 7,426 men in the 1972 Ancel Keys' study that sparked BMI’s modern-day usage in research and medical settings [2].

A WHO expert consultation concluded that for the proportion of Asians, a high risk of type 2 diabetes mellitus (DM) and CVD is significant at BMIs lower than the existing WHO overweight cut-off point (>=25 kg/m^2). Further research is required to better understand the relationship between BF% and BMI in Asian populations to properly determine cut-offs for disease risk, particularly metabolic disorders [12]. Sakurai et al. found that when using the dual-energy X-ray absorptiometry to measure BF%, Asians had significantly higher BF% than African Americans and whites with similar BMI [13]. Asian populations have been shown to exhibit a "normal" BMI, yet have significantly large WCs [14]. Differences in fat mass and fat-free mass (FFM) between UK-born South Asians and White Europeans in infancy may exist, as demonstrated by the 2012 Stanfield cross-sectional study that concluded characteristic differences in body composition were observed between the two groups in early infancy. It is the first study to show data that South Asians have reduced FFM in infancy compared to White Europeans [15]. In the 2002 Deurenberg study comparing different population groups of Asians, ranging from Indonesians, Singaporean Chinese, Malays, Indians, and Hong Kong Chinese, all Asian populations had a higher BF% at a lower BMI compared to Caucasians. It was concluded that this high BF% at low BMI can be partly attributed to differences in body build, muscularity, and height, and that universal cut-off points may not be an accurate way to estimate BF% and the risks associated with a high BF% [16]. Still, large differences between BF% and BMI existed within these Asian populations, thus generalizations surrounding Asian patients and their BF% and BMI should not be made and further research is required.

It is important for further research to be conducted regarding the potential increased risks of metabolic disease and DM for Asian populations while at a lower BF% than other, more highly-studied populations, such as Caucasians. These studies show that BMI may not accurately represent Asian populations’ risk of CVD and IR [12,14-16]. A normal BMI does not negate the increased risk of metabolic disease due to excessive abdominal fat, thus using BMI as an indicator of health in Asian populations may be more harmful than beneficial [8,9]. Physicians must be aware of this, because their Asian patients may suffer if they do not get the preventive counseling and early treatment that they need, simply because they fall into the “normal” BMI category.

Likewise, it is also not beneficial for patients to be labeled as “overweight” due to their BMI, despite having increased muscle mass and less central adiposity than other patients who fall in the “normal” BMI category. This has been observed in studies including non-Hispanic Black (NHB) individuals [17]. In the Flegal study that examined the BMI of 8,821 children and adolescents of varying racial and ethnic groups, NHB girls had significantly higher BMIs than non-Hispanic White (NHW) girls; however, the prevalence of adiposity was not significantly different between the two groups. High adiposity in NHB children was about one-half of the prevalence in the NHW, Hispanic, and Asian children in the intermediate BMI category, showing considerable differences by race-ethnic group [17]. BMI was not an accurate representation of the adiposity of NHB girls, as their BMIs were consistently higher than NHW girls, yet there was no real difference in adiposity between the two groups. These categories may not only be inaccurate depictions of adiposity but can be especially harmful to young girls growing up who already face many issues with body image. If the BMI-to-adiposity ratios were different in the two groups, yet they were still using the same BMI classification to assess their health, this classification may not be an accurate representation of the relationship between one or both groups’ weight and disease risk.

The Boston Area Community Health Survey investigated the role of biogeographic ancestry (BGA) and adiposity in 1,726 men and women using ancestry informative markers, BMI, percent body fat (PBF), and WHR. They observed positive associations between West African ancestry and cross-sectional BMI and PBF, concluding that West African ancestry may contribute to a high prevalence of total body adiposity among African Americans [18]. The All of Us (AoU) Research Program demonstration project used BMI, WC, WHR, and alanine aminotransferase (ALT) to assess racial, ethnic, and gender differences in obesity and body fat distribution in 88,195 NHW, 40,770 NHB, 35,640 Hispanic, and 5,648 Asian participants. Their results showed that BMI was highest among NHB women and lowest among Asian women and that the largest gender difference existed in NHB, whereby NHB women had higher BMI than NHB men. After the exclusion of heavy drinkers, men exhibited higher levels of ALT than women in all racial/ethnic groups [19]. This study illustrates the differences in BMI and ALT between various ethnic groups and sexes, further supporting the need for a more updated, accurate, and inclusive anthropometric index of obesity, disease risk, and mortality for all ethnic groups.

Sex Considerations

No subjects of the female sex were used in Keys’ 1972 study, yet the same BMI cut-offs for the classification of weight status and disease risk for both men and women are still being used [2,5]. The current BMI scale is likely an inaccurate representation of women’s health status and disease risks, as various factors contribute to substantial differences in CVD risk and mortality between men and women. There is considerable evidence for sex differences in cardiac autonomic modulation, sex hormones, cytokines, and lipid and glucose metabolism. The protective effects of estrogen on glucose homeostasis and greater IR in men compared to women should be examined further, as these things may play important roles in CVD, DM, and other disease risks [20]. Sex steroids also play a role in adipose tissue function. They modulate tissue responses to other hormones like insulin and catecholamines [21]. Due to differences in sex steroids among other factors, the distribution of fat differs in men and women. Women more often store fat subcutaneously and in the gluteofemoral region, while men are more likely to store excess fat in the abdominal region [7]. Interestingly, the storage of excess calories in gluteofemoral fat and subcutaneous fat (SAT) may be linked to a favorable metabolic profile, so it is important to note the proportions of each type of fat a patient carries as physicians assess patients due to these metabolic differences that may exist between abdominal and gluteofemoral fat [6].

Xin Song’s 2014 prospective cohort study studied 23,629 men and 21,965 women to estimate CVD mortality in relation to obesity and gender. Men were found to have higher CVD mortality than women in all four BMI categories (<25.0, 25.0-29.9, 30.0-34.9, and >=35.0 kg/m^2), with similar findings observed for abdominal obesity defined by WC, WHR, or WHtR [20]. In the 2015 cross-sectional study that examined 762 men and 732 women to investigate the value of SAT and visceral adipose tissue thickness (VAT) for prediction of gallstone disease (GSD), differences in the best anthropometric predictors of GSD existed between men and women as well. In men, the only indicator of obesity that showed a significant association with GSD was WHR, while WHtR, WHR, VAT, and BMI were significantly associated with GSD risk in women [22]. In a church-based cross-sectional study with 912 participants from various cities in southeastern Nigeria, BMI, WC, HC, and conicity index were significantly higher in females than males. The best predictors of hypertension risk were BMI, WHtR, WC, and ponderal index in this population [7]. These studies display the importance of reassessing our use of BMI in the healthcare setting for various groups, especially women, as there are significant differences in disease prediction accuracy between men and women.

In 2021, the All of Us (AoU) Research Program studied differences in obesity and fat distribution across gender and race/ethnicity in 88,195 NHW, 40,770 NHB, 35,640 Hispanic, and 5,648 Asian participants by comparing BMI, WC, WHR, and ALT. Consistent with previous data, the large gender disparity between NHB participants was replicated: NHB women had significantly higher BMI and rates of obesity compared to NHB men. Also consistent with previous conclusions, ALT was significantly higher in men compared to women and was lower in NHB participants compared to other racial/ethnic groups [19]. This further illustrates the physiological differences between men and women. In addition, further research is required to understand the extensively replicated and significant gender disparity between BMIs of NHB men and women.

Based on the numerous physiological and hormonal differences between men and women, it is no surprise that our disease risks and disease presentations are variable: So why do we still use the same BMI classifications? While men may have lower body fat, their risk of metabolic disease and other disease risks associated with increased visceral adiposity will not be represented properly if we continue to use the BMI. Likewise, the fundamental difference in the location where women preferentially hold their excess body fat does not properly reflect the obesity and disease risk of women if the same cut-off points as men are used. Using an anthropometric index that is better representative of men’s mortality and disease risk could be harmful to female patients because it could result in delayed diagnoses and treatment, and ultimately, increased mortality.

Promising Prospects

Moving forward, clinicians and researchers should consider the limitations associated with BMI before adopting its usage, potentially resulting in the removal of the BMI from the EMR and its replacement with an alternative anthropometric index that is more representative of abdominal fat and BF%. While this review does not offer one definitive replacement anthropometric index, there are several that exist that have been studied that may be considered superior to BMI in terms of accuracy, ability to predict CVD risk, or associations with mortality (Table 1). This may be attributed to the fact that these incorporate a waist measurement, which corresponds with abdominal adiposity.

**Table 1:** Different anthropometric indices and their corresponding formulas WHR = waist-to-hip ratio; WHtR = waist-to-height ratio; WC = waist circumference; HC = hip circumference; NC = neck circumference; ABSI = a body shape index.

Anthropometric index	Formula
BMI	(weight in kilograms/height in meters)^2^
WHR	WC/HC
WHtR	WC/height in centimeters
NC	NC
ABSI	WC/(BMI(2/3) x height(1/2))

In the SOON cohort, which examined anthropometric indices to identify fat distribution associated with high cardiometabolic risk in 305 mostly Caucasian women living in France with severe obesity, BMI was only linked to obstructive sleep apnea syndrome (OSAS). Neck circumference (NC) and WC were significantly associated with hypertension, DM, and OSAS. NC was found to be an excellent anthropometric marker of all cardiometabolic risk factors, including functional liver tests, in very obese patients. This is very clinically important because it can be difficult to obtain an accurate WC of severely obese patients, so NC may be an easy and accurate alternative. WHR was observed as a reliable marker for the identification of hypertension, DM, and other cardiometabolic markers, but not OSAS [23].

A body shape index (ABSI = WC/(BMI (2/3) x height (1/2))), a new anthropometric index that combines WC, BMI, and height, expresses excess risk from high WC. In a US population of 14,105 adults, ABSI appeared to be a substantial risk factor for premature mortality, and its correlation with mortality hazard held across the range of age, sex, and BMI, for both White and Black ethnicities, but not for Mexican ethnicity [24]. The association of BMI, WC, WHtR, WHR, and ABSI with total, cardiovascular, and cancer mortality by using Cox proportion hazard models was assessed in 2,626 men and 3,740 women from the Rotterdam study, a prospective population-based cohort from the Netherlands. In this study, ABSI was not significantly correlated with BMI, but it was strongly correlated with WC, WHtR, and WHR. ABSI had the strongest association with total, cardiovascular, and cancer mortality in comparison with the other measures [25].

In one of the first studies involving more than 300,000 adults of various ethnic groups to compare BMI and the discriminatory power of WHtR and WC in distinguishing adults with hypertension, DM, dyslipidemia, metabolic syndrome, and general CVD outcomes, WHtR had significantly great discriminatory power compared with BMI. WHtR also showed to be significantly better than WC in DM, hypertension, CVD, and all outcomes. For metabolic syndrome in particular, the difference between indices was not significant for men, but BMI was significantly poorer than WHtR among women [26]. Pasdar et al. examined the accuracy of WC, BMI, WHR, and WHtR in predicting CVD events in 10,065 Kurdish adults aged 35-65 years. WHtR served as the most simple and effective predictor of CVD events in this population, although it was noted that the precision and not the accuracy of WHR for predicting cardiac events was displayed in this study, so it may be useful as a screening tool, not a diagnostic tool. WHtR may be an excellent replacement for BMI when showing information about obesity status, as it also considers height, sex, and race differences [8].

In a meta-analysis of 38 cross-sectional and two cohort studies examining the discriminatory capacity of BMI, WC, and WHR for CVD in a total of 137,256 adults aged 18 years or older, indices of abdominal obesity, especially WHR, were shown to better predict CVD occurrence. It should be noted that most of the studies were conducted in Asian countries [27].

Limitations and gaps in this literature review

This review is not a comprehensive account of the differences in the accuracy of anthropometric indices between varying races, ethnic groups, and sexes. It states that statistically significant differences exist when comparing the use of BMI and other anthropometric indices in evaluating disease risk and mortality among various groups. This review also does not offer one clear suggestion for a replacement of the BMI but rather offers several potential replacements; however, further research regarding accuracy between varying groups, and relationship to mortality, CVD, and IR is necessary before declaring an alternative anthropometric index to replace BMI. Further research regarding the accuracy of these new anthropometric indices and their association with obesity, mortality, and cardiometabolic risks in these groups and various populations is required.

## Conclusions

As it is widely used in the medical setting, the origin and history of BMI must also be widely known and should not be overlooked by healthcare providers who choose to use this anthropometric index when evaluating and treating patients. It is imperative that we question and explore the origins and processes behind our current guidelines and practices, as they may have roots in racism and sexism, or they may not offer the best clinical accuracy in predicting disease outcomes. The BMI incorporates an individual's weight and height but does not take any abdominal measurements into consideration, which is a notable disadvantage because abdominal adiposity has been associated with increased comorbidities. As healthcare providers, we must consider each patient as a whole and consider how factors such as sex, race, ethnicity, and socioeconomic status - to name a few - each affect the health, disease risk, and life expectancy of individual patients. This review sheds light on the origins and history of BMI and sparks an important conversation regarding its clinical accuracy and how it may differ across various groups. Conducting further research in alternative anthropometric indices to find a superior replacement that considers height, sex, and race differences; accounts for abdominal adiposity, which is highly associated with cardiometabolic risk; and accurately predicts the relationship between obesity, mortality, and diseases such as CVD, DM, IR, and hypertension would be a step toward providing more inclusive, patient-centered care to the individuals that we have the privilege of taking care of as clinicians.
